# Soliton microcomb based spectral domain optical coherence tomography

**DOI:** 10.1038/s41467-020-20404-9

**Published:** 2021-01-18

**Authors:** Paul J. Marchand, Johann Riemensberger, J. Connor Skehan, Jia-Jung Ho, Martin H. P. Pfeiffer, Junqiu Liu, Christoph Hauger, Theo Lasser, Tobias J. Kippenberg

**Affiliations:** 1 Swiss Federal Institute of Technology Lausanne (EPFL), Laboratoire d’optique biomédicale (LOB), Lausanne, CH-1015 Switzerland; 2 Department of Electrical Engineering, École Polytechnique de Montréal, Montreal, QC Canada; 3 Institute of Physics, Swiss Federal Institute of Technology Lausanne, Lausanne, CH-1015 Switzerland; 4 Carl Zeiss Meditec AG, Rudolf-Eber-Straße 11, Oberkochen, 73447 Germany

**Keywords:** Lasers, LEDs and light sources, Solitons, Imaging and sensing

## Abstract

Spectral domain optical coherence tomography (OCT) is a widely employed, minimally invasive bio-medical imaging technique, which requires a broadband light source, typically implemented by super-luminescent diodes. Recent advances in soliton based photonic integrated frequency combs (soliton microcombs) have enabled the development of low-noise, broadband chipscale frequency comb sources, whose potential for OCT imaging has not yet been unexplored. Here, we explore the use of dissipative Kerr soliton microcombs in spectral domain OCT and show that, by using photonic chipscale Si_3_N_4_ resonators in conjunction with 1300 nm pump lasers, spectral bandwidths exceeding those of commercial OCT sources are possible. We characterized the exceptional noise properties of our source (in comparison to conventional OCT sources) and demonstrate that the soliton states in microresonators exhibit a residual intensity noise floor at high offset frequencies that is ca. 3 dB lower than a traditional OCT source at identical power, and can exhibit significantly lower noise performance for powers at the milli-Watt level. Moreover, we demonstrate that classical amplitude noise of all soliton comb teeth are correlated, i.e., common mode, in contrast to superluminescent diodes or incoherent microcomb states, which opens a new avenue to improve imaging speed and performance beyond the thermal noise limit.

## Abstract (web summary)

Superluminescent diodes, that provide a broadband spectrum are typically used in spectral domain coherence tomography. Here, the authors use chipscale silicon nitride resonators to generate soliton microcombs with a lower noise flor that could substitute the diode sources.

## Introduction

First demonstrated in 1991 by Huang^1^, optical coherence tomography (OCT) has become an important technique for non invasive imaging of biological tissues^2,3^. Today, OCT is a standard diagnostic tool in ophthalmology and has been extended to intravascular imaging^4^ and brain imaging^5–7^. Over the past decade, frequency domain methods (FD-OCT), i.e., spectral-domain OCT (SD-OCT) and swept-source OCT (SS-OCT), have superseded time domain OCT through their higher sensitivity^8–12^. Since, light sources and detectors for FD-OCT (both SD and SS-OCT) have improved, providing low noise, larger bandwidths and faster acquisition rates. Recently, sources comprised of a set of discrete frequencies have been proposed for FD-OCT, as they offer an increased depth-sensitivity^13,14^, reduced power exposure while maintaining a high axial resolution^15^. Most importantly, the periodicity in the tomogram offered by this novel acquisition source provides an extended imaging range through so-called optical-domain subsampling^16^, and enables significantly extending the OCT imaging range in a data efficient manner. Overall, such comb-like illuminations show great promise for imaging of non-planar samples, frequent in intra-operative scenarios^17^.

One promising implementation of such comb sources for SD-OCT are soliton microcombs. First discovered in 2007, these microcombs are generated by the nonlinear conversion processes inside microresonators^18–20^. Through adjustment of laser power and detuning, a dissipative Kerr soliton (DKS) state can be excited, providing coherence lengths and bandwidths comparable to continuous-wave and femtosecond lasers, respectively^21^. The spectrum of a DKS state consists of fully coherent laser lines with linewidths equal to the CW pump laser linewidth (typically  ~100 kHz), resulting in kilometer scale coherence lengths. Its overall spectral bandwidth can be tailored via dispersion engineering^22^ and can reach up to octave-spanning coverage^23^. In addition to their spectral properties, recent advances in fabrication technology have significantly reduced the power requirements for DKS generation, thus allowing for direct integration with semiconductor pump lasers^24,25^. Altogether, through their exceptional optical properties and wafer-scale fabrication, DKS microcombs are promising candidates as sources for imaging and in particular OCT. Here, we demonstrate the potential of microcombs as a source for OCT imaging. We first characterize the microcombs and show that bandwidths exceeding those of commercial superluminescent diodes (SLD) are possible, with overall better noise properties (especially for certain acquisition speeds). We also analyzed the noise properties of each comb tone, and show that, in contrast to incoherent frequency combs, the noise between the DKS’s comb teeth are strongly correlated. Lastly, we demonstrate the capabilities of the source by imaging ex vivo mice brain slices, and highlight its potential for circular ranging^16^.

## Results

### Dissipative Kerr solitons as a source for SD-OCT

**Fig. 1:**
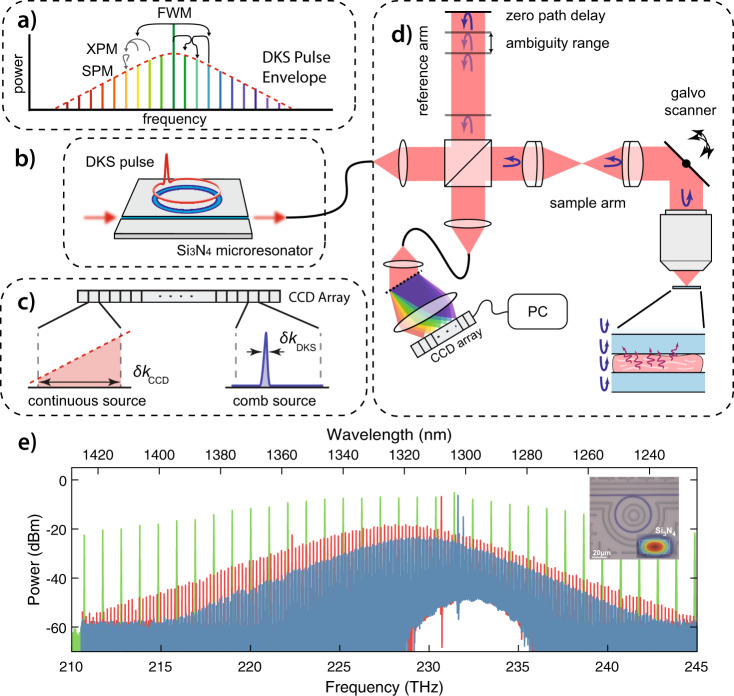
The principle of dissipative Kerr soliton enabled spectral domain optical coherence tomography (OCT). **a** A dissipative Kerr soliton (DKS), based on the system shown in **b**, where a continuous-wave laser drives nonlinear frequency conversion, through four-wave mixing (FWM), cross-phase modulation (XPM) and self-phase modulation (SPM) in a photonic chip-based Si_3_N_4_ microresonator. Here, the generated pulse train is comprised of discrete and equally spaced frequency components as determined by the free spectral range of the non-linear cavity. In particular, this process creates a frequency comb via the dual balance between non-linearity and dispersion on one hand, and loss and gain on the other. Eventually, the discrete components of this microresonator frequency comb (or continuous source as in traditional OCT) are dispersively projected onto a charged-coupled device (CCD) array as shown in **c**, after passing through a standard OCT setup as seen in **d**. Experimental data for a variety of free spectral ranges (Green 1 THz, Red 200 GHz, and Blue 100 GHz) typical of microresonator DKS are shown in **e**, along with an inset microscope photograph of a  ~1 THz microresonator, and a scanning electron microscopy photograph of a typical bus waveguide in Si_3_N_4_.

We designed novel microcombs sources for OCT imaging operating in the second optical window (NIR-II), at 1300 nm, for its relatively low water absorption and reduced tissue scattering properties. We fabricated three Si_3_N_4_ resonators (one shown in the inlet of Fig. 1e)) following the established photonic Damascene process^26^ with free spectral ranges (FSR) of  ~100, 200 GHz and 1 THz, respectively (Fig. 1 e). Through their large waveguide cross sections, the resonators achieve anomalous group velocity dispersion (GVD) in the NIR-II imaging window (see *Microresonator fabrication* in the Methods and Supplementary Note 1 for details). A microcomb, as shown in Fig. 1a), is generated by the nonlinear frequency conversion processes inside a microresonator^19^. The mutual interplay between (non-)degenerate four-wave mixing processes (FWM) and self- and cross-phase modulations (SPM and XPM respectively) provides an optical gain to the resonator modes adjacent to the pumped mode. The Kerr comb generation is achieved by sweeping the pump laser frequency from the effective blue-detuned to a defined point at the effective red-detuned side of the selected cavity resonance. For DKS comb generation, the laser sweeping typically stops at a multi-soliton state and proceeds to a single soliton state through a backward frequency tuning technique^27^.

**Fig. 2:**
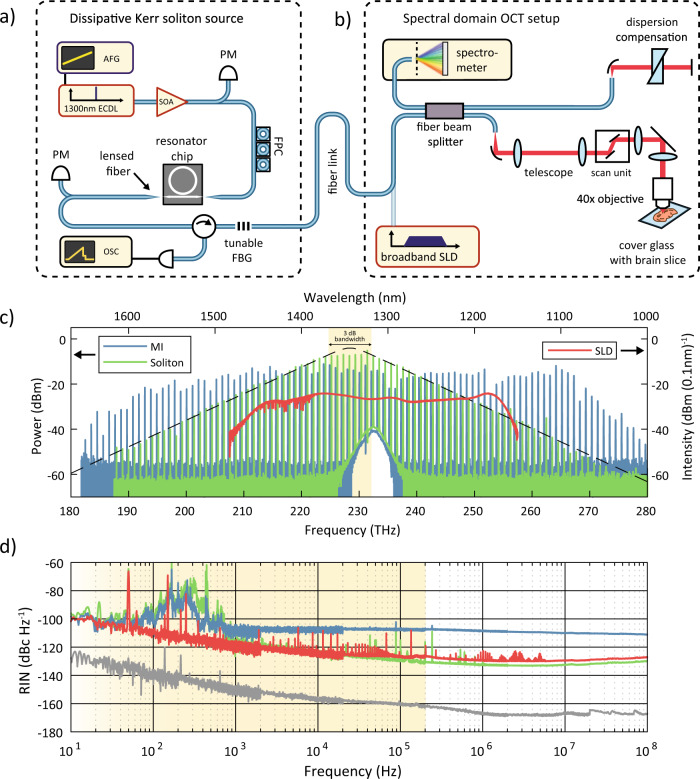
Experimental demonstration of dissipative Kerr soliton enabled SD-OCT. **a** Setup for dissipative Kerr soliton (DKS) frequency comb generation based on a 1300 nm external cavity diode laser (ECDL) amplified by a semiconductor optical amplifier (SOA). The laser wavelength is tuned by a voltage ramp provided by the arbitrary function generator (AFG) and monitored by power meters (PM). After coupling to the chip using lensed fibers, the transmitted light intensity is displayed on an oscilloscope (OSC) and provides information about the tuning process. A tunable fiber Bragg grating (FBG) is used to suppress the pump light before sending the generated light over a fiber link to the OCT setup located in a different laboratory on the campus. **b** SD-OCT setup based on a fiberized interferometer with a dispersion compensated reference arm and a high-resolution spectrometer. The setup was designed for use with a broadband SLD and the DKS comb signal was inserted without further modification for imaging. **c** A chaotic modulation instability comb (blue) and a DKS state (green) exhibiting spectral bandwidths comparable to the commercial superluminescent diode (orange). The DKS spectrum follows the characteristic sech^2^ profile and has a low density of avoided modal crossings. **d** The associated relative intensity noise (RIN) of the Kerr combs and the superluminescent diode (SLD). Note that the two Kerr comb states were generated in different resonators, as detailed in the Methods section. Here, the yellow shaded region represents the frequencies of interest for OCT measurements.

As illustrated in Fig. 2, the nonlinear frequency conversion bandwidth of the 1 THz microcombs can readily reach and exceed the bandwidth of SLDs. This is demonstrated for two distinctly different operational Kerr frequency comb states: the DKS and the chaotic modulation instability (MI) states (shown in Fig. 2 c). The DKS state, shown in green, exhibits a characteristic sech^2^ spectral envelope and reaches a spectral coverage similar to the reference SLD source. The cross section of the 1 THz DKS waveguide, 780 × 1450 nm^2^, provides an anomalous GVD (*D*_2_/2*π* ~ 40 MHz) for soliton pulse formation. The 3 dB bandwidth of the DKS spectrum, highlighted in Fig. 2 c), is ~8.3 THz, corresponding to a 38 fs transform limited pulse.

The chaotic Kerr comb state, shown in blue in Fig. 2c, provides a spectral coverage well exceeding the SLD’s, due to the lower GVD (*D*_2_/2*π* ~ 20 MHz) originating from its smaller microresonator cross section (730 × 1425 nm^2^). The resulting spectral envelope is overall flat but, in contrast to the DKS state, exhibits local power variations caused by avoided mode crossings.

### Noise characteristics of soliton microcombs

To assess the noise characteristics of these novel sources and their applicability to OCT imaging, we first measure their relative intensity noise $RIN=\frac{{S}_{P}(f)}{\langle {P}^{2}\rangle }$, with *S*_*P*_(*f*) denoting the single sided power spectral density of the intensity fluctuations (shown in Fig. 2d), and demonstrate that while the MI state provides a broader spectral coverage, its chaotic nature results in an increase in RIN of nearly 20 dB, extending to very high offset frequencies in the GHz domain (Fig. 2d)^28^. These measurements were performed for different FSRs (i.e., 100, 200 GHz and 1 THz), and resulted in similar RIN profiles between resonators (data not shown here). Accordingly, although chaotic comb states in a Si_3_N_4_ microresonator have been demonstrated in OCT imaging^29^, their higher noise should ultimately limit OCT performance as compared to SLDs, especially at elevated imaging speeds. Meanwhile, we also show that the DKS soliton state has comparable intensity noise with the SLD, at frequencies higher than 10 kHz. In the low frequency regime, mechanical modes of the input and output lensed fiber-coupling result in a broad noise peak spanning from 100 to 1000 Hz for both the MI and DKS states, which can be mitigated through optimized packaging or feedback loops.

Even more so, the ultimate performance limit of coherent sources at high offset frequencies, such as the DKS comb, is given by the photon shot noise ($RIN=\frac{2\hslash \omega }{P}=-145$ dBc Hz^−1^ with 20 μW power on the detector) and improves with optical power. In contrast, in the case of broadband, incoherent light sources, the RIN is limited by spontaneous emission beat noise^30,31^ (*RIN* = 1/*B*_0_ = −136 dBc Hz^−1^ for a 45 THz rectangular bandwidth SLD source), which ultimately limits the dynamic range gain with high source powers in the reference arm^32^.

**Fig. 3:**
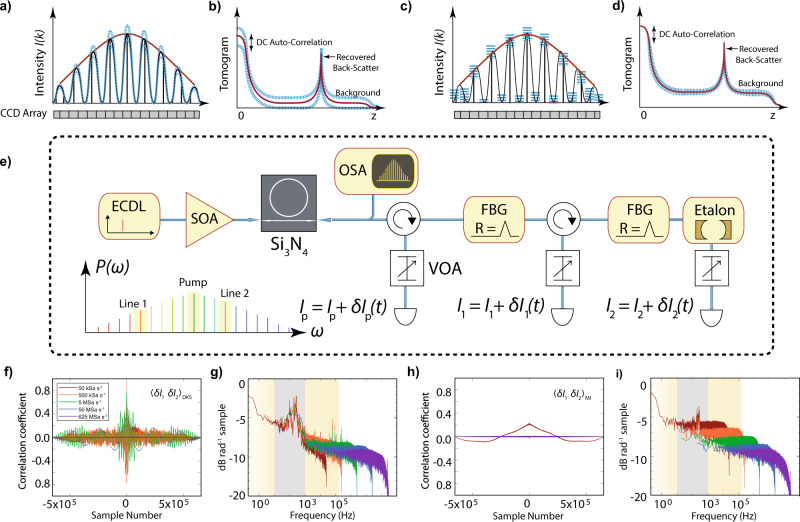
Frequency dependent noise correlations. **a** A frequency comb interferogram (black) is dispersively projected onto a charged-coupled device (CCD) array and its correlated intensity noise (blue) modulates the full comb envelope (red). **b** Upon Fourier transformation, the tomogram’s DC peak is sensitive to the noise, but no change in background signal-to-noise ratio and dynamic range occurs. **c** In the case of uncorrelated intensity noise between various comb lines, each line of the interferogram exhibits uncorrelated noise, which manifests in the tomogram seen in **d** as an increase in background signal. **e** Setup for intensity noise correlation measurement: the source, comprising an external cavity diode laser (ECDL), a semiconductor optical amplifier (SOA), the microchip and an optical spectrum analyser (OSA) generates dissipative Kerr soliton (DKS) and modulation instability (MI) states by laser piezo tuning. Various lines are then filtered from the resulting spectrum using fiber Bragg gratings (FBG), sent through variable optical attenuators (VOA), and sampled with a high-resolution oscilloscope. **f**, **h** The cross correlation of lines "1" and "2" for the DKS and MI states, where lines "1" and "2" correspond to 1272 nm and 1320 nm, respectively. The *x*-axis denotes the relative lag in units of the inverse sampling rate, as derived from the samples per second (Sa s^−1^), with all color indications shared between sub-figures. **g**, **i** Depict the cross power spectral densities corresponding to **f** and **h**, respectively. In the spectral plots, the gray shaded region highlight technical noise, likely originating from acoustic modes of the input- and output coupling fibers, while the yellow shaded region indicates the range of typical SD-OCT A-scan rates.

Next, we explore one unique feature of soliton microcombs; the high-degree of coherence between individual comb lines. This is especially important in the context of OCT, as line-by-line intensity noise of the frequency comb’s retrieved spectra (Fig. 3 a, c) corresponds to pixel-by-pixel noise in the retrieved image (Fig. 3b, d). Indeed, as an image in SD-OCT is produced via a Fourier transform of the interferogram, only uncorrelated intensity noise between various pixels degrades the final image^33^. Noise in the amplitude of the spectrum’s envelope will act only on the DC component of the tomogram (Fig. 3a, b), whereas uncorrelated intensity fluctuations between the different optical frequencies will lead to a higher noise level at all depths of the tomogram (Fig. 3c, d). To investigate these intra-tone noise properties, we performed the cross-correlation of intensity fluctuations on pairs of comb lines, using the experimental setup described in Fig. 3e^34^. From both DKS or MI combs, individual comb lines are filtered and time traces are recorded and cross-correlated (Fig. 3f, g) for various sampling speeds. The corresponding cross power spectral densities (PSD) are depicted in Fig. 3g, i. In practice, we chose two lines, at 1272 nm and at 1320 nm (lines 1 and 2 respectively in Fig. 3). In the DKS state, we observe a peak correlation coefficient between the two chosen lines of approximately 0.95, corresponding to a sampling rate of 500  kSa s^−1^. The maximum correlation coefficient near zero lag stays well above 0.8 for sampling frequencies up to 5  MSa s^−1^, indicating that intensity noise between DKS comb lines is highly correlated even at elevated frequencies. In contrast, for the fully developed MI state, the maximum correlation coefficient between lines 1 and 2 is approximately 0.24, and occurs for the lowest sampling speed (50 kSa s^−1^). For all higher sampling frequencies, however, the correlation coefficient decreases to approximately 0.01, indicating highly uncorrelated intensity noise between comb lines, We expect a similar behavior for the the classical noise of nearly all incoherent sources, including SLD sources.

As mentioned earlier, given that the ultimate limit of the noise properties of frequency domain OCT is set by the degree of correlation of intensity noise between various spectral channels^33^, and therefore different optical frequencies, the DKS state can offer significant advantages, in terms of noise, as compared to the MI state. In view of these differences in noise performances, as well as the DKS’s superior nonlinear efficiency and bandwidth, we chose to use a DKS source for the OCT experiments presented here.

### Spectral characteristics of microcombs for OCT imaging

In frequency domain OCT, depth-resolved information about the sample is conveyed through the amplitude and frequency of an interferogram. A reflectivity profile is obtained through a Fourier transform of the recorded spectrum on the spectrometer. From sampling theory, the maximum imaging depth obtainable ${z}_{\max }$ is therefore dictated by the spectrometer’s spectral resolution *δk*_CCD_ as^35^:

1 $$\pm\! {z}_{\max }=\pm\! \frac{1}{4\delta {k}_{{\rm{CCD}}}}$$

As such, OCT systems designed for high axial resolution and deep penetration imaging require a detection capable of registering a broadband spectra at a fine spectral resolution. In practice, combining these two features is cumbersome in SD-OCT due to the limited length of current array detectors (typically between 1024 and 2048, and exceptionally 8196 pixels^36^), ultimately limiting either the effective resolution or the available imaging range.

When comb-like sources, such as Kerr combs, are employed instead of a continuous spectrum, the discrete set of frequencies will generate a periodicity in the tomogram if the frequency/time difference between the combs is sampled by the detector^16^. The frequency of this periodicity, called the ambiguity range, is determined by the source’s repetition rate *f*_rep_ (which also corresponds to the temporal separation between the individual pulses). For Kerr combs, the repetition rate is given by the microresonator FSR (D_1_/2*π*):

2 $${z}_{{\rm{ambiguity}}}=\frac{c}{2{n}_{{\rm{tissue}}}}\frac{1}{{f}_{\!{\rm{rep}}}}\approx \frac{c}{2{n}_{{\rm{tissue}}}}\frac{2\pi }{{{D}}_{{\rm{1}}}}$$

with the speed of light *c* and the tissue refractive index *n*_tissue_. For the imaging experiments carried out in this work, we used microresonators with a 1 THz FSR, leading to an ambiguity range of ~71 μm compared to a maximum imaging range of ~2 mm offered by the spectrometer. In contrast, the lower FSR DKS sources shown in Fig. 1e offer repetition rates down to 100 GHz, corresponding to an increased ambiguity range of  ~710 μm.

In addition to their discreteness in frequency, DKS sources also possess interesting temporal coherence properties. Although the overall coherence length of the source is dictated by its bandwidth, the coherence length of each comb tone of the DKS source equals that of the driving pump laser and thus amounts to several kilometers for a pumping linewidth around 100 kHz. As mentioned earlier and highlighted in Eq. (1), the attainable imaging range in FD-OCT is typically dictated either by the spectral resolution of the spectrometer or by the width of the swept spectral line (for spectral-domain and swept-source respectively). When combining DKS sources with an SD-OCT system, a mismatch can therefore occur between the imaging range (given by the spectrometer, here ~2 mm) and the coherence length of each comb tone (here  >2 km). As such, the coherence lengths reached here largely exceed the imaging ranges of typical OCT systems, entailing novel advantages and disadvantages for imaging, which will be detailed in the Discussion.

### OCT imaging with a DKS microcomb

**Fig. 4:**
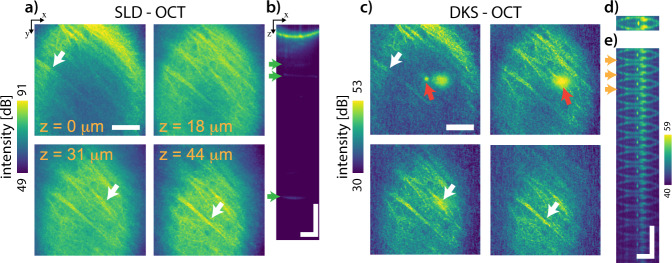
Qualitative performance comparison of sources for ex vivo cerebral tissue imaging. En-face images at different depths of a slice of brain tissue were obtained with both a superluminescent diode (SLD) and a dissipative Kerr soliton (DKS) source **a** and **c** respectively, revealing the presence of highly scattering neuronal fibers (pointed by blue arrows). The en-face views obtained with the DKS source also contain additional features, pointed by red arrows, such as bright vertical stripes, circular ring patterns, and higher intensity regions. The cross-sections for both SLD and DKS imaging **b** and **d**, respectively, highlight the imaging’s field curvature, highly reflective structures below the sample (pointed by white arrows) and the presence of an ambiguity range when imaging with the discrete DKS source (pointed by red arrows). Scalebars: 50 μm (**a** and **c**), 100 μm (**b**, **d** and **e**).

The difference in performance between the SLD and the DKS as sources for OCT imaging was qualitatively assessed by imaging a ~50 μm thick slice of a mouse brain tissue. The OCT was equipped with a 40 ×  0.8 NA objective (Olympus) to obtain a lateral resolution of ~1.5 μm and a depth-of-field shorter than the source’s ambiguity range. In a first step, we imaged the slice using the SLD source, providing an axial resolution of ~6 μm in air. Figure 4a presents en-face views over a 200 × 200 μm^2^ area at specific depths, whereas panel b shows the cross-section images of the SLD based OCT tomogram. These views present similar features as other OCT observations of cerebral tissues^7^, such as neural fibers (pointed by white arrows), which appear as directional, bright, and fine structures over dim neuropil. Within the neuropil, darker circular structures seemingly point to the presence of neuronal cell bodies, as already observed in high resolution OCT^7,37^.

Secondly, without modifying any imaging parameters nor touching the sample, the SLD was disconnected from the system and replaced with the DKS source, providing an axial resolution of ~10 μm in air. Figure 4 c, d and e show the OCT tomogram of the same sample with the DKS light source. The neural fibers can be clearly observed in the en-face views with higher contrast. However, the neuropil appears darker and fewer details can be discerned. Additional artifacts, as indicated by the red arrows, are present in some of the DKS views and are likely caused by the combination of two characteristics of the DKS source: its discrete set of frequencies and its narrow linewidth. Overall, the dynamic range obtained in the DKS images is reduced by  ~19 dB compared to the SLD. This discrepancy could originate from the significantly lower power provided by the DKS source (estimated to be up to a fourth of the SLD power) and from the presence of the spurious back-reflections, ultimately drowning the collection of weakly scattering features. For both sources, the A-scan rate was maintained at 46 kHz. The images presented in panels c and d were obtained by selecting only the comb tones from the interferograms, dismissing non-illuminated pixels. Conversely, for panel f the entire recorded interferogram was used. More details on the processing are available in Supplementary Note 3 The axial resolution of the SLD and the DKS were extracted using a reflective mirror (as shown in Supplementary Fig. 3) and are ~6 μm and  ~10 μm, respectively.

As mentioned earlier, the frequency discretization of the source will lead to a periodic image folding along the axial dimension, as similarly observed by Siddiqui et al.^16^. The ambiguity range of the source can be observed in the cross-section (Fig. 4e) and manifests itself as an axial periodicity of the structures (orange arrows in Fig. 4e). As the comb width is significantly narrower than the spectrometer’s spectral resolution, the coherence length of the DKS comb tones exceeds both the ambiguity range and the spectrometer’s imaging range. The aforementioned image folding and extended coherence length thus allows reflections within the optical path to interfere with the reference arm, and will ultimately be superimposed with the features under investigation. As a result, some of the artifacts in the DKS images might stem from the folding of structures beyond the DKS’s ambiguity range, such as reflections from optical components and the coverslide (illustrated in Fig. 1d) or from the back-scattering of cerebral structures. Some of the artefacts pointed by red arrows in Fig. 4c) can be observed at deeper locations in the SLD’s tomogram, highlighted by green arrows in Fig. 4b). Typically, these strong reflections will occupy a significant portion of the spectrometer’s dynamic range and could ultimately drown the fine details of the image, as previously observed in OCT^38,39^.

## Discussion

In this manuscript, we have demonstrated the use of a DKS source for SD-OCT. We show that such soliton sources (DKS) are an interesting candidate for SD-OCT imaging through their low-noise, discrete set of frequencies and large bandwidths. Our work highlights the outstanding noise performance of the source: specifically, the DKS (a coherent broadband source), equals and even outperforms an SLD (fully incoherent source) in its relative intensity noise (RIN). Equally important, DKS feature a unique property, in our knowledge previously unseen in OCT sources: the noise between the comb tones comprising the soliton frequency combs shows an unprecedentedly high degree of correlation.

This feature is particularly important in OCT, as images are obtained through a Fourier transform of the spectrum. As such, noise common to all comb tones does not degrade the dynamic range, whereas relative uncorrelated fluctuations from pixel to pixel contribute to a significant dynamic range reduction^33^. Therefore, in the context of SD-OCT, each DKS comb line represents a narrowband laser with Poissonian photon statistics. Technical noises appear correlated on all comb lines and hence our findings indicate that the established SD-OCT design requirement of vanishing photon degeneracy factor^40^$\delta =\frac{FWC}{\delta \nu \cdot \tau }\gg 1$, where FWC denotes the full-well capacity of the CCD-detector, *δν* the spectrometer resolution, and the integration time *τ*, does not apply to DKS sources, which potentially allows for longer range, higher resolution and increased dynamic range SD-OCT systems.

With the noise of the source characterized, we imaged ex vivo mice fixed brain slices, and found that the novel source allows for visualization of similar features to an SLD source, although with an overall reduced dynamic and imaging range. Overcoming these pitfalls can be achieved by optimizing both the OCT instrument and the source. First, the artefacts present in Fig. 4c–e could be suppressed either by using solely reflective optical elements^41^ or through a dark-field implementation^38,39^. As these spurious reflections can occupy a significant portion of the dynamic range of the camera, eliminating these features could help further enhance the system’s imaging capabilities. Second, the ~71 μm ambiguity range available with 1 THz DKS is too short for most imaging applications. It is however sufficient for imaging of thin flat tissues and for certain optical biopsy applications^42–52^, wherein there is need for a real-time assessment of brain and tumor tissue on a cellular level, as patient survival has been shown to be correlated to the extent of tumor resection^53^. Moreover, as shown in Fig. 1e, DKS sources with shorter FSRs down to 100 and 200 GHz are already available with similar noise profiles as the one used here for imaging. These sources would enable reaching ambiguity ranges up to ~0.7 mm, which are compatible with most in vivo imaging applications^16^. Third, fully exploiting the circular ranging capabilities of the source requires reading the interferograms in a complex-valued form^17^, which can be attained by adding acousto-optic frequency shifters to the system^54,55^. Fourth, in the presented implementation, the DKS sources provide lower powers than commercial SLDs and swept-source lasers, which could limit their applicability. Nevertheless, the technology to produce DKS sources for OCT is still novel, and strategies to develop higher power DKS sources are under investigation. Lastly, the central wavelength of 1300 nm used here is not suitable for all in vivo applications, especially human ophtalmology. Nevertheless, the source’s design can be modified, enabling shifting of the central wavelength to shorter spectral ranges, such as 1 μm, as demonstrated previously^56,57^.

Overall, in addition to the unprecedented noise performance of the DKS source and the increased imaging efficiency available through optical-domain subsampling, frequency combs could potentially alleviate certain shortcomings of SD-OCT detection schemes by facilitating *λ*-to-k mapping and reducing depth dependant sensitivity roll-off^13,14^. As demonstrated earlier, the DKS source used for OCT imaging here provides an axial resolution of ~10μm in air, which is similar to most commercial SLDs and swept-source lasers. Nevertheless, DKS sources could also lead to higher axial resolutions at 1300 nm: as highlighted in Fig. 2c, the power spectral density of the DKS source exceeds the SLD’s from ~1250 nm to 1500 nm. As such, using spectral shaping, the DKS could provide a bandwidth comparable or larger than current broadband SLDs used for 1300 nm imaging.

The high performance of the DKS source could lead to a significant miniaturization of the OCT system. The optical-domain sub-sampling capabilities of the source, highlighted in Fig. 4c, already indicate a potential shortening of the reference arm of ~2 mm. Furthermore, although not demonstrated here, the long coherence length of the DKS combs could enable further shortening of the length of the reference arm, reducing the instrument’s footprint. In traditional SD-OCT systems, the path delay difference between the reference and sample arms needs to be smaller than the maximum imaging range of the spectrometer to record an interference. In the case of a frequency comb, this condition is alleviated through optical sub-sampling, so long as the path delay difference is within the coherence length of each line of the source. As the DKS source used in this study has a theoretical coherence length for each comb line beyond a kilometer, the reference arm length could be significantly shortened, ultimately paving the way to future miniaturized and potentially more efficient high-resolution OCT imaging systems. Lastly, the optical-domain subsampling properties of our source would be highly valuable in human in vivo imaging, wherein the sample geometry is often non-planar and features could exceed the imaging range, such as in ophtalmology and intra-operative OCT. Altogether, the aforementioned noise and spectral properties of DKS microcombs hint to their significant unexplored potential for future exploitation in SD-OCT.

## Methods

Here we describe the experimental realization of Kerr comb based SD-OCT. Figure 2 shows the experimental setting consisting of two distinct setups located in buildings spaced by about ~700 m. A fiber link connects the setup for DKS generation and the SD-OCT setup between the two laboratories.

### Microresonator fabrication

The samples employed are 1 THz FSR microresonators formed by Si_3_N_4_ waveguides. Figure 1e shows the microresonator used in this work. These resonators were fabricated using the photonic Damascene process which avoids common processing challenges of thick Si_3_N_4_ films^26,58^ and has recently allowed for microresonator Q factors exceeding 10 million^24^. The continuous wave pumping light is coupled into the Si_3_N_4_ chips via a double inverse taper^59^. For the 1 THz DKS comb, the cross section is 1.45 × 0.78 μm^2^ while for the chaotic comb, it is 1.425 × 0.73 μm^2^. The bus waveguides (design width 0.55 μm for DKS and 0.525 μm for the chaotic comb) couple the light into the ring resonators (22.71 μm radius) are mode matched to excite the fundamental TM_00_ mode. The resonance linewidth is below 100 MHz as has been measured in the recent publication^60^. The waveguide cross-section of the 100 and 200 GHz FSR DKS microresonators is 1.52 × 0.82 μm^2^. The simulated dispersion profiles, including the modal dispersion (*D*_2_) and the modal deviation from the resonance frequency of the nearest mode (*D*_int_) can be found in Supplementary Fig. 1.

### Kerr comb generation

The DKS light source is pumped by a 1300 nm external cavity diode laser, which is amplified up to  ~650 mW power using a semiconductor optical amplifier (SOA). The amplified light is coupled to the silicon nitride microresonator chip via lensed fibers. The pump polarization can be adjusted via a paddle controller and both the power before and after the chip are monitored via power meters (PM). We estimate a soliton excitation power in the bus waveguide of ~290 mW. An arbitrary function generator (AFG) provides the voltage ramp signal driving the laser frequency tuning. A standard voltage ramp tuning method^27^: through the voltage-ramp tuning, a multi-soliton state is excited which is then converted into a single soliton through backward tuning. A tunable fiber Bragg grating (FBG) is used to attenuate the residual pump light while the back-reflected pump is detected through a fast photodiode and shown on an oscilloscope (OSC) to monitor the laser tuning. The generated DKS spectrum is free of avoided mode crossings causing strong local power deviations typically originating from the multimodal nature of the waveguide.

### Noise and noise correlation measurements

Noise measurements of DKS, MI and SLD sources as presented in Fig. 2 have been performed using a New Focus 1811 photodetector. The optical power on the photodetector was attenuated to 20 μW in order to avoid the detector saturation threshold of 50 μW. Special care was applied to minimize spurious noises from the photodiode bias power supply and other technical detector noises. RIN spectra were recorded using a logarithmic sweep of resolution bandwidth (RBW) and frequency span to reduce measurement time. The full optical spectrum of DKS, SLD or MI was used for measurement. Noise correlation measurements were conducted by filtering the pump and two separate comb lines using an fiber-coupled Etalon filter and a fiber Bragg-gratings in conjunction with optical circulators. The suppression of residual pump light and of neighboring comb lines was better than 25 dB in all cases. The intensity noise spectra were recorded using the same photodetectors as in case of the RIN measurements and the signals were sampled using a 500 MHz digital oscilloscope in “high-resolution” mode, i.e. applying a low-pass filter at the Nyquist-frequency. Again a logarithmic sweep of sampling rates was performed to determine correlations across a wide spectral bandwidth. The cross-correlation coefficient at zero delay hence measures the mean degree of correlation of all noise components up to the Nyquist frequency of a certain sampling rate. Oscillations of the correlation coefficient indicate dominant sources of correlated noise related to fiber-chip coupling with lensed fibers. Although correlated technical noise is also present in case of the MI comb, the overall correlation is low because the excess thermal noise is already dominant at low frequencies.

### OCT imaging

The generated DKS comb is then sent to a custom-built OCT setup through a ~700 m long optical fiber link (to connect the source from one laboratory to the OCT setup in another laboratory). The SD-OCT setup was designed for a commercial SLD with a central wavelength *λ*_0_ = 1310 nm and bandwidth *δλ* = 150 nm (LS2000C, Thorlabs) and its detection is based on a highly sensitive spectrometer, as described previously^61^. Both the source and detection are connected via a broadband fiber beam splitter (TW1300R5A2, Thorlabs) with a dispersion compensated reference arm and a sample arm comprising a galvo mirror scan unit (6210H, Cambridge Technologies), a high NA objective (LUMPLFLN-40XW, Olympus) and imaging optics. The scanner control and data readout are performed by a connected computer with a high-speed input. The output optical power of the SLD source is ~9.15 mW while the DKS comb is <2.3 mW. All images were acquired at an A-scan rate of 46 kHz. The post-processing steps, including k-space resampling and Fourier transformations were performed using a custom software implemented in MATLAB (Mathworks). The axial resolution of the SLD and DKS systems were characterized by placing a mirror in the front focal plane of the objective and were measured as ~6 μm and ~10 μm in air respectively.

### Image processing

The images presented in Fig. 4 were obtained after Fourier transform of the spectral interferograms recorded by the spectrometer. Prior to visualization, the dynamic range of the data was reduced using first a logarithmic operation (10 × log_10_()) and a clipping operation (same operations for both DKS and SLD images). The data was then spatially smoothed using a median filter in MATLAB^62^, planes at different depths were selected. The clipping limits were obtained by taking the 0.01% and 99.9% intensity values of the imaged planes, after median filtering.

Background subtraction was performed, prior to Fourier transforming, by averaging each spectra of a B-scan into a single background vector, which was then subtracted to the entire B-scan. This step was repeated for each B-scan of the volume, essentially acting as a high-pass filter on the data with a cut-off frequency around ~90 Hz. This step was performed for both SLD and DKS data. The DKS data was processed in two separate ways, as shown in Fig. 4, either by considering the entire interferogram or by selecting only the comb peaks. In the first processing method, the entire interferogram was considered, including non-illuminated pixels. The obtained A-scan for each position is of the same length as the spectral interferogram (Fig. 4e). In the second method, the comb tones positions on the interferogram were first identified by computing the local maximal value around the tone. Using their positions, shorter interferogram were obtained by eliminating all other pixels (non comb tone pixels). The resulting A-scan were therefore significantly shorter than those obtained in the first method, and include solely one ambiguity range (Fig. 4c, d). The dynamic range of the planes for both methods was 19 and 23 dB for the first and second processing pipelines respectively.

### Brain tissue preparation

All animal procedures were carried out according to Swiss regulations under the approval of the veterinary authority of the canton of Vaud (protocols VD3056 and VD3058), are in-line with the 3Rs and follow the ARRIVE guidelines. After transcardiac perfusion, the brains of B6SJL/f1 mice were extracted, placed into 4% PFA overnight and then placed in a solution of 30% glucose. The brains were finally cut into slices of ~50 μm using a microtome and placed on a glass coverslide. These samples had been prepared for previous studies^63,64^, no new samples were prepared for this manuscript.

## Supplementary information

Supplementary Information

## Data Availability

The data and codes used to produce the plots within this paper are available at 10.5281/zenodo.4399060.
