# Supplementary material for: Soliton microcomb based spectral domain optical coherence tomography
Source: Nat Commun. 2021 Jan 18;12:427. doi: 10.1038/s41467-020-20404-9 (PMC7813855; doi:10.1038/s41467-020-20404-9)
Supplement: Supplementary file 1 — Supplementary Information [file 41467_2020_20404_MOESM1_ESM.pdf]

Supplementary Information
Soliton microcomb based spectral domain optical coherence tomography

Marchand et al.

SUPPLEMENTARY NOTE 1

The dispersion profiles, including the GVD parameter ($D_2/2\pi$) and the integrated dispersion ($D_{\text{int}}/2\pi$) (Supp. Fig. 1) of the microresonators were simulated using COMSOL multiphysics® with the 2D axial symmetric model. The cross-section dimension is $1.45 \times 0.78\,\mu\text{m}^2$ for the DKS comb and $1.425 \times 0.73\,\mu\text{m}^2$ for the chaotic comb. Both the radius of the resonators are $22.71\,\mu$m. The TM_{00} mode in the waveguides was calculated with 2D axial symmetry, which accounts for the additional normal dispersion due to the tightly bent waveguide. The smaller cross-section of thhe

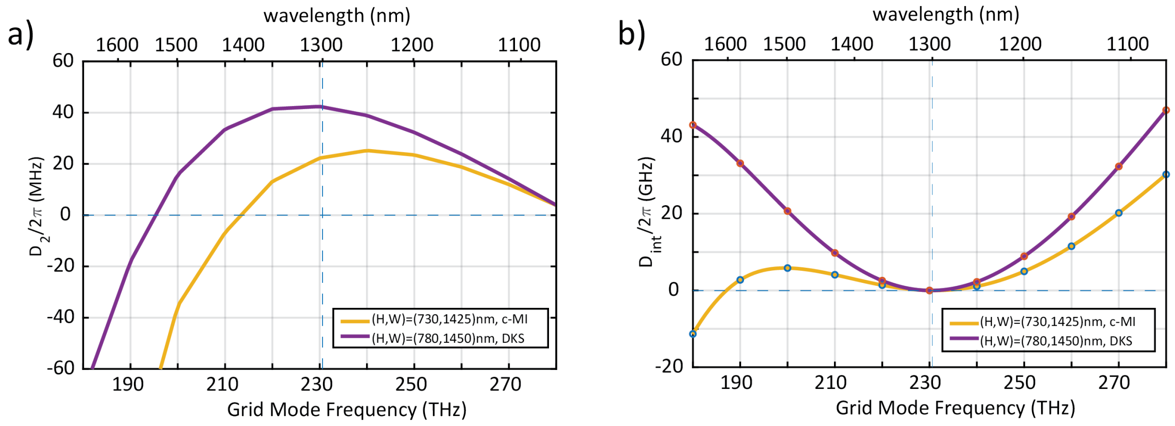

Supplementary Figure 1. **Dispersion simulations of the microresonators** a) group velocity dispersion (GVD) parameter ($D_2/2\pi$) and b) integrated dispersion ($D_{int}/2\pi$) of the microresonators were simulated using COMSOL multiphysics® simulation package with the given dimension parameters detailed in the Methods section

SUPPLEMENTARY NOTE 2

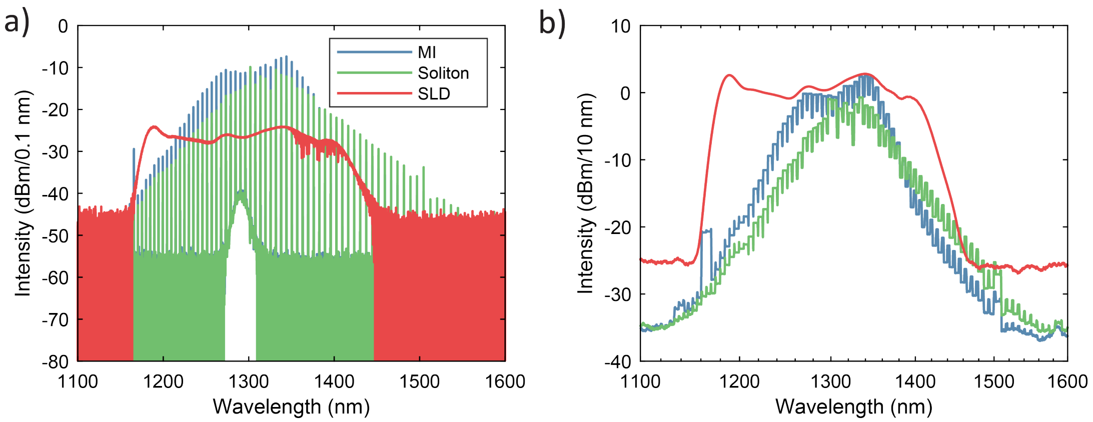

Supplementary Figure 2. **Optical power spectral densities of solitons and chaotic Kerr combs in the sample capable of soliton generation.** a) Optical intensity spectra of modulation instability state (MI), soliton state and superluminescent diode (SLD) measured with resolution bandwidth 0.1 nm. b) Same spectra, but with 10 nm resolution bandwidth.

Supplementary Figure 2 shows the optical spectra of the different source types that are compared in this work with different resolution bandwidth. Averaging the resolution bandwidth to 10 nm, we see that the superluminescent diode source (LS2000C, Thorlabs), which actually combines two independent SLD diodes exceeds the per pixel power of our soliton microcombs. The chaotic comb matches the per pixel power of the SLD but at the cost of increased RIN noise. The same sample (780 nm x 1450 nm) is chosen here for both chaotic modulation instability comb and soliton

microcomb generation.

SUPPLEMENTARY NOTE 3

To characterize the imaging performance of the DKS as a light source for OCT imaging, we used a highly reflective mirror substrate as a sample. Supplementary Figure 3 a) shows the DKS signal as recorded by the spectrometer's image sensor without the mirror (i.e. signal from the reference arm). Interestingly, although the line width is significantly shorter than the camera's spectral sampling, the comb line can be sampled on adjacent pixels as can be seen in the inset. From the Gaussian-like shape of the recorded peak, we believe this effect to be likely caused by the diffraction limited size of the spot on the camera. Other potential causes include electronic cross-talk between the pixels and a sub-optimal matching between comb teeth spacing and the detector pixel pitch. The apparent divergence between this observation and the conceptual illustration presented in Fig. 1 a) does not however impact the circular ranging abilities of the source, as the coherence length of a single comb tone still exceeds the imaging range of the spectrometer. After placing the mirror, we obtain the tomogram shown in logarithmic scale in Supplementary Fig. 3 b) by Fourier transforming the signal obtained from the spectrometer. A resolution of $\sim 10\,\mu$m and an ambiguity range of $\sim 71\,\mu$m are derived in the air. The signal attenuation over depth observed, also termed roll-off, is caused primarily by the spectrometer's finite spectral sampling despite the fine width of the comb's lines.

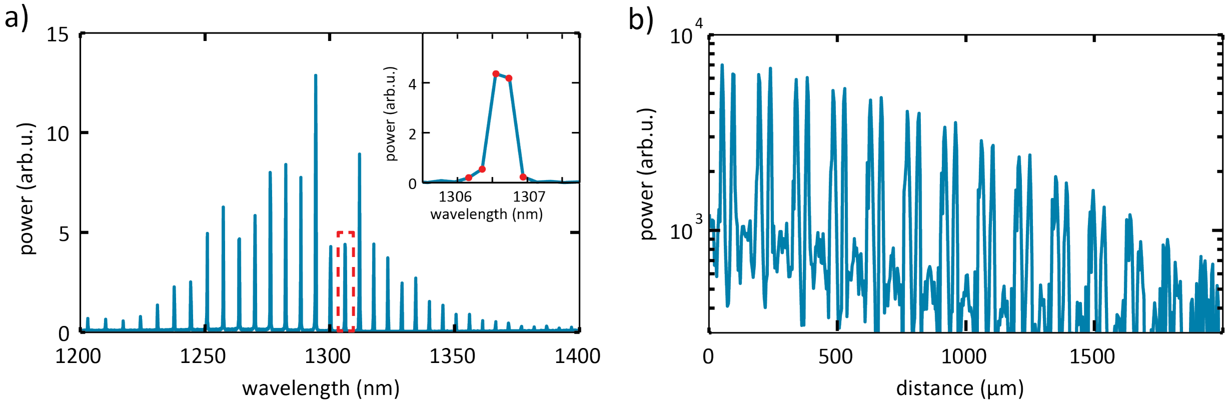

Supplementary Figure 3. **Characterization of imaging performance.** a) Disspative Kerr soliton (DKS) comb spectrum as acquired by the spectrometer's line sensor. Inset showing a zoomed-in view of a single tooth of the comb being sampled by adjacent pixels. b) Tomogram of the a mirror placed under the objective obtained with the DKS source, obtained after re-sampling and Fourier transformation of the spectral interferogram.
